# In vitro and molecular modeling insights into α-amylase inhibition by tamarind seed-derived trypsin inhibitor: Implications for hyperglycemic control

**DOI:** 10.1371/journal.pone.0333289

**Published:** 2025-09-29

**Authors:** Larissa Aida Lemos de Souza, Caio Patrício de Souza Sena, Felipe Carlos de Macêdo Oliveira, Raphael Paschoal Serquiz, Ana Júlia Felipe Camelo Aguiar, Amanda Maria de Souza Nascimento, Ana Karoliny Xavier de Gois Silva, Rose de Paiva Chaves, Anna Beatriz Santana Luz, Thaís Souza Passos, Davi Serradella Vieira, Ana Heloneida de Araujo Morais

**Affiliations:** 1 Postgraduate Program in Nutrition, Federal University of Rio Grande do Norte, Natal, Brazil; 2 Postgraduate Program in Chemistry, Federal University of Rio Grande do Norte, Natal, Brazil; 3 Postgraduate Program in Pharmaceutical Sciences, Federal University of Rio Grande do Norte, Natal, Brazil; 4 Department of Biochemistry, Federal University of Rio Grande do Norte, Natal, Brazil; 5 Postgraduate Program in Biochemistry and Molecular Biology, Federal University of Rio Grande do Norte, Natal, Brazil; 6 Nutrition Couse, Federal University of Rio Grande do Norte, Natal, Brazil; 7 Health Sciences Center, Federal University of Recôncavo da Bahia, Bahia, Brazil; 8 Department of Nutrition, Federal University of Rio Grande do Norte, Natal, Brazil; 9 Institute of Chemistry, Federal University of Rio Grande do Norte, Natal, Brazil; Kwara State University, NIGERIA

## Abstract

Inhibitors of enzymes involved in carbohydrate digestion may be a potential option for glycemic control in Diabetes Mellitus. This study aimed to evaluate the effect of the trypsin inhibitor isolated from tamarind seed (*Tamarindus indica* L.) (TTI) on α-amylase. After confirmation of the obtaining and characterization of the TTI, the in vitro inhibitory activity of the TTI against α-amylase was analyzed. The interaction of the modeled structures’ theoretical TTI (TTIp 56/287) and five of its derived peptides with α-amylase was also evaluated in silico using Docking and Molecular Dynamics, and their functional properties were examined. The Interaction Potential Energy (IPE) and the main interactions of the peptide-α-amylase complex were described using three-dimensional representations. TTI presented 100% antitryptic activity and a molecular mass of approximately 21 kDa. In vitro, inhibition of α-amylase was higher than 37%. These results were corroborated by computational analyses, which demonstrated strong interaction between the TTIp 56/287 complex and its peptides with the enzyme. The Root Mean Square Deviation (RMSD) and Root Mean Square Fluctuation (RMSF) analyses showed good stability. IPE was −705.08 kJ/mol for DTVHDTDGQVPL and −584.11 kJ/mol for TIAPACAPKPAR. Electrostatic interactions stand out, especially the salt bridge, between the main residues that interacted in the complexes (DTVHDTDGQVPL, TIAPACAPKPAR, and TVSQTPIDIPIGLPVR). Additionally, the bioactive potential predicted two candidates with good stability, a long half-life, and bioactivity in an intestinal simulation environment. This is the first report of tamarind trypsin inhibitor or its peptides inhibiting α-amylase. Thus, the amino acid sequences DTVHDTDGQVPL and TIAPACAPKPAR were revealed as candidates that could be tested for action against α-amylase and possibly for glycemic control.

## 1. Introduction

Obesity is considered a chronic non-communicable disease (NCD) and has become a global public health problem [1]. Lifestyle (diet and physical activity) is one of the main factors that influence the progression of obesity [2]. Additionally, this condition can lead to excessive fat storage and an increase in adipose tissue, which leads to a moderate chronic inflammatory process, which triggers a range of complications, including insulin resistance (IR), type 2 Diabetes Mellitus (T2DM), cardiovascular diseases, cancer, and hepatic steatosis, among other related diseases [3].

Diabetes Mellitus (DM) affects people of all ages, and over the years, the number of cases worldwide has been increasing dramatically. By 2045, it is estimated that 783 million people worldwide will have the disease, affecting approximately 1 in 8 adults, including type 1 DM (T1DM) and T2DM, as well as other unclassified types of Diabetes [4]. T1DM and T2DM are the most common types related to the absence of insulin production and gradual loss of adequate insulin secretion, respectively. This fact results in difficulty in controlling blood glucose [5]. Maintaining control of DM has been one of the most significant challenges, and therefore, making regular physical activity and a healthy diet are necessary for satisfactory blood glucose control [6].

Nevertheless, drug therapy is often indispensable. On the other hand, undesirable effects, such as nausea, abdominal distension, diarrhea, and liver damage, which can be caused by the use of a variety of therapeutic agents, are consolidated in the treatment of DM, such as acarbose, voglibose, and miglitol [6,7]. This drives the search for less aggressive alternatives, preferably natural and with a low risk of toxicity [7]. From this perspective, dietary proteins stand out as excellent sources of bioactive peptides. These peptides, which are products of protein proteolytic hydrolysis, have been highlighted in the food and health industries as they demonstrate versatility in treating diseases [8,9]. Therefore, identifying peptides with these properties may be a promising strategy for treating DM.

Considering that the increase in blood glucose, common in T2DM, is related to the carbohydrate digestion process, enzyme inhibitors involved in this process have also been evaluated to control or reduce the glycemic response [10]. Among the protease inhibitors, the trypsin inhibitor isolated from tamarind seeds has been the target of research conducted by the Nutrition and Bioactive Substances for Health (NutriSBioativoS) research group at UFRN, Brazil, in various forms, including partially purified (TTI), purified (TTIp), and nanoencapsulated (ECW) forms. It has shown bioactive potential in preclinical studies [11] and significant effects on blood glucose control [12,13].

Regarding TTI, Medeiros et al. [14] determined the amino acid sequence of this inhibitor, as well as its three-dimensional structure definition. In the same study, the theoretical model of TTI, number 56, conformation number 287 (TTIp 56/287), demonstrated the best stability, which allowed the performance of in silico analyses. Subsequently, in silico analysis revealed that TTI and its derived peptides have great potential for treating T2DM as they interact with the insulin receptor (IR) [12,14]. Furthermore, in a preclinical study, TTI reduced fasting blood glucose levels in Wistar rats with T2DM [12]. However, it was unclear how TTI acted, and it remains unknown whether it can inhibit α-amylase, a recognized therapeutic target for the treatment of T2DM. Although studies on plant-derived α-amylase inhibitors exist [9,10], the use of trypsin inhibitors extracted from tamarind seeds (*Tamarindus indica* L.) remains little explored in the literature.

In this context, in silico studies have emerged as a promising tool for identifying and validating therapeutic targets, as well as discovering bioactive molecules with a wide range of health applications [12]. The study combines in vitro assays with computational modeling (docking and molecular dynamics), which strengthens the robustness of the results and aims to prospect peptides derived from TTI. Most studies in this area are limited to basic laboratory tests or computational modeling alone; few integrate both in a complementary manner. This study investigated the possibility that TTI and/or its peptides inhibit α-amylase in vitro and interact with it in silico, a recognized therapeutic target for controlling T2DM. To the best of our knowledge, this is the first report of tamarind trypsin inhibitor or its peptides inhibiting α-amylase.

## 2. Methodology

### 2.1. Obtaining trypsin inhibitor from tamarind seeds (TTI)

The tamarind fruit (*Tamarindus indica* L.) was purchased from a local store in Natal/RN, Brazil, and registered in the National System for Genetic Heritage Management and Associated Knowledge (SisGen) under the number AF6CE9C. The TTI was obtained in accordance with the recommendations of Carvalho et al. (2016) [15].

The Bradford (1976) [16] methodology was used for protein quantification. The test was performed in triplicate to evaluate the inhibition against trypsin, as described by Kakade, Simons, and Liener (1969) [17]. To assess the degree of purity and estimate the molecular mass of the CE (crude extract), F1 (Fraction 1), F2 (Fraction 2), and TTI proteins, the methodology developed by Laemmli (1970) [18] was used.

### 2.2. In vitro inhibition of α-amylase

The inhibitory activity of TTI against α-amylase was performed according to the methodology initially described by Telagari and Hullatti (2015) [19] and adapted by Oliveira et al. (2024) [20]. In a 96-well microplate, a reaction mixture containing 15 μL of α-amylase (0.125 mg/mL), 30 μL of sodium phosphate buffer (0.1 M, pH = 6.9) and 30 μL of samples (at concentrations of 0.3, 0.6 and 1.5 mg/mL) was added, which were pre-incubated at 37 °C for 20 minutes. After preincubation, 30 μL of 0.125% soluble starch in 0.1 M sodium phosphate buffer (pH 6.9) was added as a substrate, and the mixture was incubated for another 30 min at 37 °C. Then, 75 μL of 3,5-dinitrosalicylic acid solution was added and heated at 100 °C in a water bath for 10 min. After adding 150 μL of water, the absorbance was measured at 540 nm. In the negative control, samples were not added, and the reaction volume was completed with the same buffer used in the test. Three acarbose solutions (at the same concentrations as the samples, 0.3, 0.6, and 1.5 mg/mL) were used as positive controls. The concentration range was based on literature [19,20]. The percentage of α-amylase inhibition was calculated using Equation 1.


$$\text{In vitro inhibition of }\alpha -\text{amylase }(\%)\;=\;(\text{1}\;-\;{\text{abs}}_{\text{sample}}/{\text{abs}}_{\text{control}})\;x\;\text{1}\text{0}\text{0}$$ (1)

### 2.3. In silico study of the interaction of TTIp 56/287 and its derivative peptides with α-amylase

#### 2.3.1. Molecular docking between TTI 56/287 and α-amylase.

The interaction between the trypsin inhibitor isolated from tamarind seeds (TTI) and the α-amylase enzyme was analyzed computationally in this study. For this purpose, the theoretical model number 56 and conformation number 287 of TTI (TTIp 56/287) were used [14]. The molecular model of α-amylase was obtained from the RCSB Protein Data Bank, PDB ID 5VA9 [21].

Molecular docking was performed to predict the preferred conformations of the ligand molecules [TTIp 56/287 in the binding site of the target macromolecule (α-amylase)]. The binding site that anchors the inhibitors was defined based on the PDB ID 5VA9 structure obtained by Goldbach et al. (2019) [21]. Molecular docking was performed to predict the preferred conformations of the ligand molecules [TTIp 56/287 in the binding site of the target macromolecule (α-amylase)]. The binding site that anchors the inhibitors was defined based on the PDB ID 5VA9 structure obtained by Goldbach et al. (2019) [21]. Docking was conducted with the High Ambiguity Driven Biomolecular DOCKing server (HADDOCK 2.4), which has been extensively applied to protein–peptide systems [https://doi.org/10.1002/prot.25802]. The resulting complexes were ranked according to the HADDOCK Score (HS), a weighted combination of van der Waals, electrostatic, desolvation, and restraint violation energies, together with a buried surface area term. Complexes with lower HS values were considered to represent more favorable binding poses and were selected for further analyses [22].

Conformational models of the TTIp 56/287 and α-amylase interaction were derived from ten molecular docking simulations, systematically exploring the enzyme’s binding site by varying the ligand’s amino acid sequence. The objective of this strategy was to contemplate the surfaces with the greatest steric probability of coupling occurring.

#### 2.3.2. Obtaining, predicting, modeling, and selecting three-dimensional structures of peptides derived from TTIp 56/287.

Peptides were generated *in silico* by enzymatic cleavage of the protein sequence reported by Gomes et al. (2024) [23], using trypsin and chymotrypsin virtual digestion via the Peptide Cutter tool (https://www.expasy.org/resources/peptidecutter). The molecular structures of the resulting peptide sequences were modeled using the Pep-Fold3 server [24]. The three-dimensional structures of the peptides containing five or more amino acid residues were refined and optimized through molecular dynamics simulations using the GROningen Machine for Chemical Simulations (GROMACS) software [25], version 2023.1, implemented with the CHARMM36 force field [26] and employing the Transferable Intermolecular Potential 3 Point (TIP3P) water model [27].

Peptides were then subjected to site-directed molecular docking against the α-amylase binding site. Based on an arbitrary criterion, the three peptides with the best HS scores were selected for subsequent molecular dynamics simulations. To complete a set of five peptides for these simulations (arbitrary choice), two additional peptides, predicted to possess antidiabetic bioactivity, were selected using the AntiDMPred computational predictor [28], a machine learning-based tool.

#### 2.3.4. Molecular dynamics simulation of TTIp 56/287-derived peptides and α-amylase.

Molecular dynamics simulations were performed using the GROningen Machine for Chemical Simulations (GROMACS) software [25], version 2023.1, implemented with the CHARMM36 force field [26]. The complex systems were solvated in a dodecahedral box using the Transferable Intermolecular Potential 3 Point (TIP3P) water model [27]. The systems were neutralized by adding Na^+^ and Cl^-^ counterions at a concentration of 0.15 mol/L. The Leap-Frog algorithm solved the motion equations [29] with an integration interval of 2.0 fs. Long-range interactions were modeled using the Particle-Mesh Ewald sum (PME) [30] with a cut-off of 1.2 nm. Van der Waals interactions were also calculated using the same limit. This value was chosen to ensure that the radius did not extend beyond the distance between the protein-ligand complex and the walls of the simulation box, which is essential for accurately capturing long-range interactions. Bonds involving hydrogen atoms were constrained using the Linear Constraint Solver for Molecular Simulations (LINCS) algorithm [31].

Systems were minimized by the steepest descent algorithm for 10,000 steps with a tolerance of 10 kJ mol − 1 nm − 1 and equilibrated in two steps of 1 ns, the first in the NVT ensemble (closed systems with constant volume and temperature) and the second in the NPT ensemble (closed systems with constant pressure and temperature). The temperature was controlled at 310.15 K (37 °C) by the V-rescale thermostat [32], while the pressure was controlled by the Parrinello–Rahman barostat [33] (for the NPT ensemble). The total simulation time in the production dynamics was 300 ns for all systems. This period of time was determined through Root Mean Square Deviation (RMSD) analysis. The simulation was run until the RMSD reached a steady state, characterized by its oscillation around a mean value without any significant upward or downward trend. A minimum stability period of 200 nanoseconds (ns) was used to ensure sufficient data for statistical analysis. The simulations were conducted using physiological conditions to model the biological system accurately.

#### 2.3.5. Analysis of molecular dynamics simulations.

The stability of the complexes was evaluated during and after the molecular dynamics simulations through Root Mean Square Deviation (RMSD), Root Mean Square Fluctuation (RMSF), and Interaction Potential Energy (IPE), as well as analysis of B-factor structures. RMSF, IPE, and B-factor were calculated from the final 50 ns of the simulation. The interaction analysis included the calculation of the IPE and a description of the main interactions: intermolecular hydrogen bonds (conventional and carbon), electrostatic (charge-charge, salt bridge, pi–cation, pi–anion), and hydrophobic (pi-pi, pi–pi-t, amide–pi, alkyl, pi–sigma and pi–alkyl).

Images of peptide-protein interactions were produced by BIOVIA Discovery Studio software and the 3D Protein Imaging server [34]. Graphs were generated by Graphing, Advanced Computation, and Exploration of Data (Grace) [33].

#### 2.3.6. Prediction of bioactivity and cell penetration capacity, half-life in gut-like environment.

Peptides selected for computational modeling with length > 10 aa (since the responsible server requires sequences longer than 10 aa) were screened for their bioactive potential using the PeptideRanker bioactive peptide prediction server [35]. Peptides with a score ≥ 0.5 in PeptideRanker were classified as potentially bioactive. The membrane translocation potential of the peptides was evaluated using the CellPPD online server [36].

The computational prediction of the half-life (MV) of the peptides was performed via the HLP server, and the time and stability were verified. Peptides with MV < 0.1 seconds were classified as ‘low stability,’ those with MV > 0.1 and < 1.0 seconds were classified as ‘normal stability’, and those with MV > 1.0 seconds were classified as ‘high stability.’

### 2.4. Statistical analysis

Data were expressed as mean ± standard deviation (SD) of three independent determinations. Statistical analyses were performed using Analysis of Variance (ANOVA) followed by Tukey’s post hoc test. Results were considered statistically significant at p < 0.05 (95% confidence level), using GraphPad Prism version 9.4.0.

## 3. Results

### 3.1. Obtaining trypsin inhibitor isolated from tamarind

**Fig 1:**
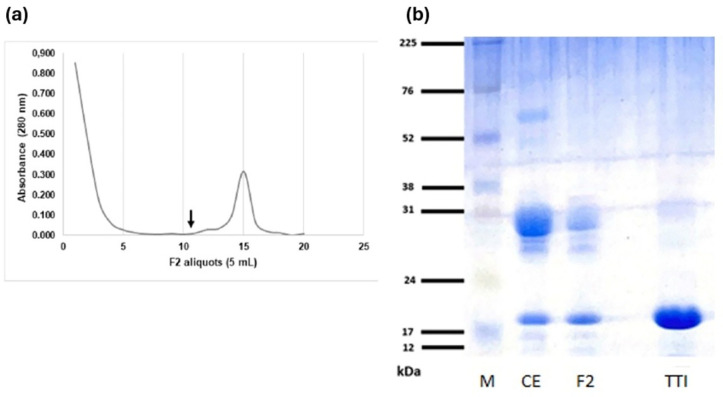
Chromatographic profile of F2 and molecular mass estimation of TTI. 35 mg of F2 were applied to Trypsin-Sepharose CNBr 4B affinity chromatography. a) The column was previously equilibrated with 50 mM Tris-HCl buffer, pH 7.5, and elution of unretained material (before the arrow) was performed with the same buffer. Proteins adsorbed on the matrix (starting from the arrow) were eluted with HCl (5 mM), and aliquots of F2 (5 mL) were monitored at 280 nm. The antitryptic activity of the protein peak was verified using 100 µL of TTI, using BapNA as substrate. b) Discontinuous and denaturing polyacrylamide gel (SDS-PAGE) at 12% stained with Comassie Blue. M: marker; CE: crude extract; F2: fraction 2 (precipitate 2 with ammonium sulfate in 30-60%). TTI: trypsin inhibitor isolated from tamarind seeds. The arrow represents the addition of HCl.

The chromatographic profile of F2 (protein fraction saturated with 30–60% ammonium sulfate) was demonstrated (Fig 1A), and the isolation of TTI was visualized on a 12% SDS-PAGE gel (Fig 1B), showing a predominant protein band of approximately 21 kDa. TTI presented 0.7 mg of proteins and 100% inhibition of trypsin (393.47 IU/mg) in 70 g of tamarind seed flour.

### 3.2. In vitro inhibition of α-amylase

**Fig 2:**
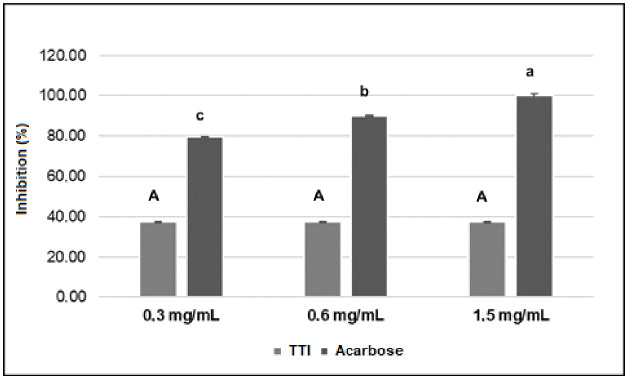
In vitro anti-α-amylase activity of TTI compared to acarbose. The anti-α-amylase activity was verified using 30 µL of TTI, using starch as substrate. TTI: trypsin inhibitor isolated from tamarind seeds. ^abc^Different lowercase letters indicate a statistical difference between the means (p < 0.05). ^ABC^Similar uppercase letters indicate no statistical difference between the means (p > 0.05).

Regarding in vitro inhibition of α-amylase, TTI, and the control drug (acarbose), a commercial synthetic α-amylase inhibitor were tested at concentrations of 0.3 mg/mL, 0.6 mg/mL, and 1.5 mg/mL. It was observed that 100% inhibition of α-amylase by acarbose occurred at the highest concentration (1.5 mg/mL). In addition, a significant reduction (p < 0.05) in inhibition was observed when the acarbose concentration was reduced. Unlike what was observed with acarbose, TTI presented lower inhibition (approximately 37.3%), with no statistically significant differences, at the three concentrations tested (Fig 2). The percentage inhibition of ITT and acarbose were significantly different (p < 0.05), and the inhibition of acarbose was higher against α-amylase.

### 3.3. In silico study of the interaction of TTIp 56/287 and derivative peptides with α-amylase

#### 3.3.1. Molecular docking TTIp 56/287 – α-amylase.

**Fig 3:**
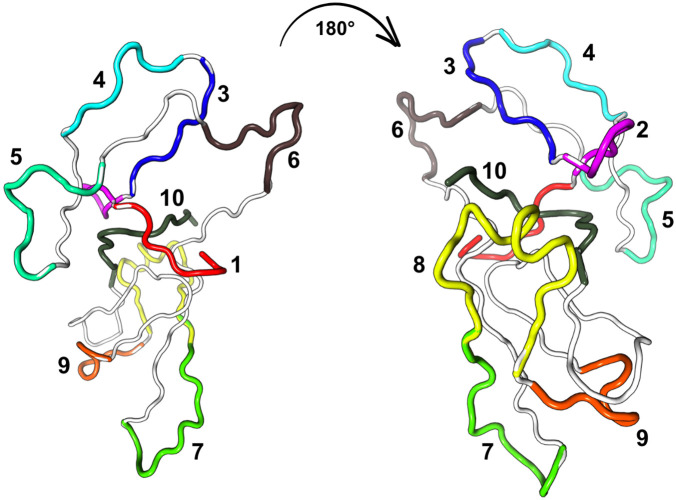
Molecular models at two viewing angles of TTIp 56/287 for molecular docking study with α-amylase (PDB ID 5VA9). The numbers indicate the amino acid sequences used in docking: 1 (DTVHDTDGQV), 2 (PLNNAGQYYI), 3 (LPAQQGKGGG), 4 (LGLSNDDDGN), 5 (PIDIPIGLPVRSF), 6 (TTALSLNIEFTI), 7 (EKGYTVPKLSDDF), 8 (SSAPFKLKQFEEDYKLVYCSK), 9 (SESGERKCVDLG) and 10 (KKVDEESSEEWSIV). TTIp 56/287: model number 56 and conformation number 287 of the purified tamarind seed trypsin inhibitor.

The results obtained through the HADDOCK 2.4 server identified the preferred conformations of the TTIp 56/287 – α-amylase complexes. Ten amino acid sequences contained in TTIp 56/287 were chosen to guide the molecular docking studies: sequence 1 (DTVHDTDGQV), encompassing residues from ASP001 to VAL010; sequence 2 (PLNNAGQYYI), from PRO011 to ILE020; sequence 3 (LPAQQGKGGG), from LEU021 to GLY029; sequence 4 (LGLSNDDDGN), from LEU031 to ASN040; sequence 5 (PIDIPIGLPVRFS), from PRO049 to SER061; sequence 6 (TTALSLNIEFTI), from THR070 to ILE081; sequence 7 (EKGYTVPKLSDDF), from GLU101 to PHE113; sequence 8 (SSAAPFKLKQFEEDYKLVYCSK), from SER115 to LYS135; sequence 9 (SESGERKCVDLG), from SER136 to GLY147; and sequence 10 (KKVDEESSEEWSIV), from LYS171 to VAL184. Thus, we have the TTIp 56/287 structure, highlighting the amino acid sequences selected for the molecular docking study with α-amylase (PDB ID 5VA9) (Fig 3).

The parameters provided by the HADDOCK Web Server for the most stable clusters of the TTIp 56/287 – α-amylase complexes were evaluated (Table 1). The number of structures obtained for each complex, the Haddock Score (HS), the RMSD, the relative energies of the van der Waals (E_VWD_) and electrostatic Coulombic (E_ELEC_) interactions, and the Z-Score were obtained. Through these data, it was observed that regions 2 (PLNNAGQYYI) and 1 (DTVHDTDGQV) presented the best HS, −151.5 ± 3.0 and −143.0 ± 4.9, respectively, and were chosen as the best fitting orientations of TTIp 56/287 – α-amylase. More negative HS values indicated stronger and more energetically favorable binding.

**Table 1:** Parameters obtained from the HADDOCK Web Server in molecular docking for ten different docking orientations for the TTIp 56/287 – α-amylase complex.

Amino acid sequence	N° of structures	HS	RMSD	E_VDW_	E_ELEC_	Z-score
DTVHDTDGQV	176	−143.0 ± 4.9	20.9 ± 0.1	−115.3 ± 9.6	−184.8 ± 39.7	−1.4
PLNNAGQYYI	194	−151.5 ± 3.0	1.0 ± 1.0	−103.9 ± 8.1	−260.1 ± 32.8	0.0
LPAQQGKGGG	163	−95.9 ± 1.4	17.0 ± 0.7	−82.3 ± 7.1	−91.4 ± 23.9	−1.6
LGLSNDDDGN	180	−89.7 ± 4.6	13.7 ± 0.2	−70.8 ± 5.5	−146.5 ± 35.2	−1.6
PIDIPIGLPVRFS	173	−94.8 ± 12.4	13.7 ± 1.1	−68.5 ± 9.3	−168.8 ± 17.1	−2.0
TTALSLNIEFTI	190	−99.3 ± 2.5	1.3 ± 0.8	−67.2 ± 4.2	−155.9 ± 27.5	−1.4
EKGYTVPKLESDDF	170	−131.4 ± 5.3	0.7 ± 0.4	−78.6 ± 4.8	−266.3 ± 28.6	−1.4
SSAAPFKLKQFEEDYKLVYCSK	180	−133.3 ± 8.4	0.6 ± 0.4	−79.0 ± 10.2	−419.2 ± 51.0	−1.9
SESGERKCVDLG	179	−90.5 ± 9.1	0.8 ± 0.5	−68.3 ± 4.5	−133.1 ± 17.7	−1.4
KKVDEESSEEWSIV	155	−137.0 ± 3.9	1.6 ± 0.9	−109.5 ± 10.2	−286.8 ± 70.5	−2.0

Number of structures obtained, HADDOCK Score (HS), Root Mean Square Deviation (RMSD), relative energies of van der Waals interactions (EVWD) and electrostatic Coulombic interactions (EELEC), and Z-score. TTIp 56/287: three-dimensional model number 56 and conformation number 287 of the trypsin inhibitor purified from tamarind seeds.

**Fig 4:**
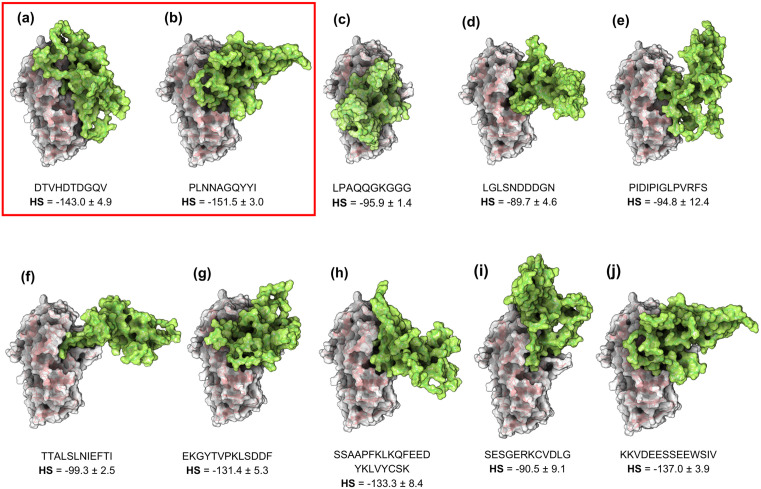
Molecular structures in the molecular surface representation of the TTIp 56/287 (green) – α-amylase (gray and red) complexes. The structures were obtained from molecular docking with orientational docking of the numbered sequences, with the first two sequences exhibiting the highest HS highlighted in red. (a) DTVHDTDGQV, (b) PLNNAGQYYI, (c) LPAQQGKGGG, (d) LGLSNDDDGN, (e) PIDIPIGLPVRSF, (f) TTALSLNIEFTI, (g) EKGYTVPKLSDDF, (h) SSAPFKLKQFEEDYKLVYCSK, (i) SESGERKCVDLG and (j) KKVDEESSEEWSIV. TTIp 56/287: model number 56 and conformation number 287 of the trypsin inhibitor purified from tamarind seeds. Letters: D—Aspartic acid, E—Glutamic acid, R—Arginine, K—Lysine, N—Asparagine, H—Histidine, Q—Glutamine, S—Serine, T—Threonine, A—Alanine, G—Glycine, V—Valine, P—Proline, L—Leucine, I—Isoleucine, W—Tryptophan, C—Cysteine. TTIp 56/287: model number 56 and conformation number 287 of the purified tamarind seed trypsin inhibitor.

The molecular model structures of the TTIp 56/287 – α-amylase complexes were generated (Table 1) in molecular surface representation (Fig 4). Regions 2 (PLNNAGQYYI) and 1 (DTVHDTDGQV) were chosen as the best-fitting orientations of TTIp 56/287 – α-amylase.

#### 3.3.2. Obtaining, predicting, modeling, and selecting the three-dimensional structures of peptides derived from TTIp 56/287.

Five of the 13 peptides evaluated were selected and used in this study and are listed with their respective Haddock Score (HS) (Table 2). This factor was decisive in the selection of the peptides since the more negative the HS value, the higher the ligand-protein affinity. The energy and scoring parameters for the most stable clusters of the peptide-α-amylase complexes were also presented (Table 2). In decreasing order of HS: peptide 1 DTVHDTDGQVPL (12 aa; HS = −79.2 ± 6.3); peptide 2 TIAPACAPKPAR (12 aa; HS = −73.1 ± 9.3); peptide 3 TVSQTPIDIPIGLPVR (16 aa; HS = −72.8 ± 3.9); peptide 4 DEQSSEK (7 aa; HS = −41.1 ± 2.2); peptide 5 ILPAQQGK (8 aa; HS = −40.6 ± 3.5).

**Table 2:** Primary data obtained from the scoring process after molecular docking for each peptide-α-amylase complex.

Peptide (Amino acid sequence)	N° of structures	HS	RMSD	E_VDW_	E_ELEC_	Z-score
DTVHDTDGQVPL	159	−79.2 ± 6.3	0.3 ± 0.2	−59.9 ± 3.1	−131.8 ± 10.3	−2.2
TIAPACAPKPAR	158	−73.1 ± 9.3	0.4 ± 0.3	−48.6 ± 7.8	−200.5 ± 9.2	−1.8
TVSQTPIDIPIGLPVR	159	−72.8 ± 3.9	0.2 ± 0.1	−63.1 ± 3.0	−133.4 ± 5.7	−1.7
DEQSSEK	154	−41.1 ± 2.2	0.8 ± 0.0	−36.6 ± 1.9	−138.0 ± 5.4	−1.8
ILPAQQGK	163	−40.6 ± 3.5	1.3 ± 0.2	−34.7 ± 3.6	−101.4 ± 17.0	−1.6

Number of structures obtained, HADDOCK Score (HS), Root Mean Square Deviation (RMSD), relative energies of van der Waals interactions (E_VWD_) and electrostatic Coulombic interactions (E_ELEC_), and Z-score of the lowest HS structure. Letters: D—Aspartic acid, E—Glutamic acid, R—Arginine, K—Lysine, N—Asparagine, H—Histidine, Q—Glutamine, S—Serine, T—Threonine, A—Alanine, G—Glycine, V—Valine, P—Proline, L—Leucine, I—Isoleucine, W—Tryptophan, C—Cysteine. The peptides are derived from the cleavage of TTIp 56/287: three-dimensional model number 56 and conformation number 287 of trypsin inhibitor purified from tamarind seeds.

The last two peptides, DEQSSEK and ILPAQQGK, were chosen using the AntiDMPred software. This tool employed machine learning to predict antidiabetic potential by selecting candidates based on a probability threshold (*pt*) optimized for specificity and sensitivity. Notably, the *pt* values for DEQSSEK and ILPAQQGK were the highest (0.59), indicating strong potential [28].

**Fig 5:**
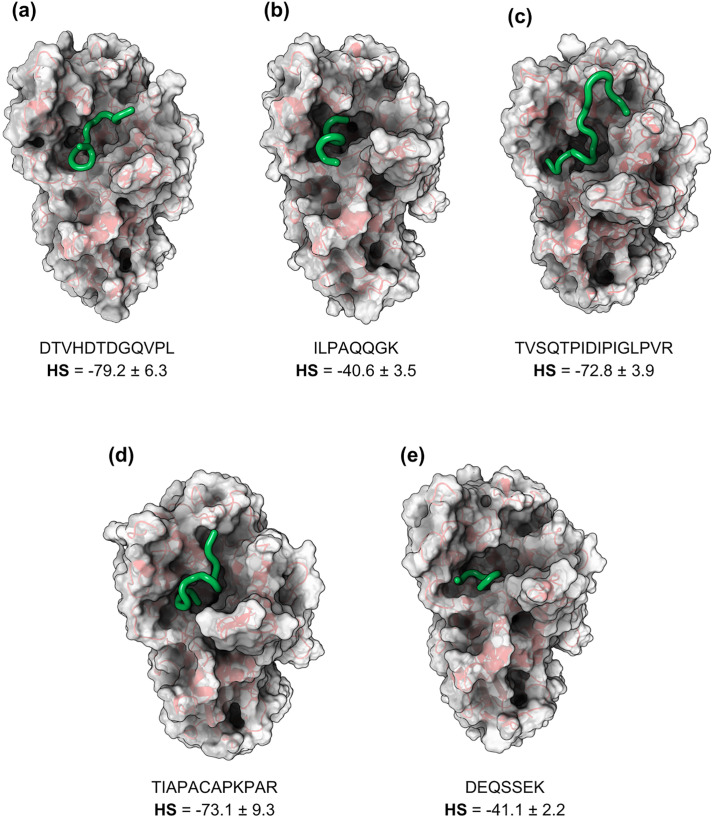
Surface and cartoon representations of the most stable conformations of the peptide-α-amylase complexes. The conformations were obtained from molecular docking for the peptides with the following amino acid sequences. (a) DTVHDTDGQVLP, (b) ILPAQQGK, (c) TVSQTPIDIPIGLPVR, (d) TIAPACAPKPAR and (e) DEQSSEK. The peptides are highlighted in green, and the α-amylase in gray with red. Letters: D—Aspartic acid, E—Glutamic acid, R—Arginine, K—Lysine, N—Asparagine, H—Histidine, Q—Glutamine, S—Serine, T—Threonine, A—Alanine, G—Glycine, V—Valine, P—Proline, L—Leucine, I—Isoleucine, W—Tryptophan, C—Cysteine. The peptides are derived from the cleavage of TTIp 56/287: model number 56 and conformation number 287 of trypsin inhibitor purified from tamarind seeds.

The structural representation of surfaces and cartoon of the peptide-α-amylase complexes were also presented (Fig 5). As previously mentioned, five peptides with the most stable conformations were selected based on molecular docking results. It was found that the first three peptides presented (DTVHDTDGQVPL, TIAPACAPKPAR, and TVSQTPIDIPIGLPVR) exhibited the best HS values (<−72) and suggested the most stable docking.

**Fig 6:**
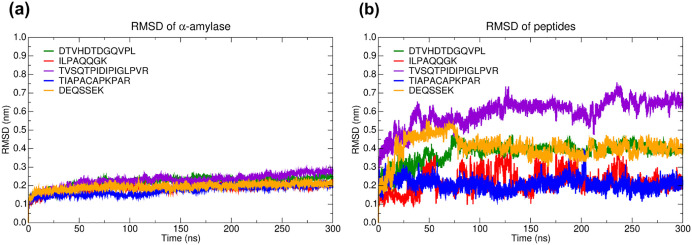
Root mean square deviation (RMSD) over 300 ns of molecular dynamics simulations of the peptides-α-amylase complexes. Different colors identify peptides, the x-axis represents the simulation time (ns), and the y-axis indicates the RMSD value (nm). Letters: D—Aspartic acid, E—Glutamic acid, R—Arginine, K—Lysine, N—Asparagine, H—Histidine, Q—Glutamine, S—Serine, T—Threonine, A—Alanine, G—Glycine, V—Valine, P—Proline, L—Leucine, I—Isoleucine, W—Tryptophan, C—Cysteine. The peptides are derived from the cleavage of TTIp 56/287: model number 56 and conformation number 287 of trypsin inhibitor purified from tamarind seeds.

Regarding the molecular dynamics results, the RMSD plot over the 300 ns simulation time (Fig 6) allowed us to evaluate the conformational stability of the complexes. Using the initial structure as a reference, the RMSD values were calculated based on the α-carbons. The RMSD analysis indicated that α-amylase maintained structural stability across all complexes, with RMSD values consistently below 2.5 nm. In contrast, the peptides exhibited notable conformational fluctuations, ranging from 0.1 to 0.7 nm, highlighting the dynamic behavior of TIAPACAPKPAR and ILPAQQGK.

**Fig 7:**
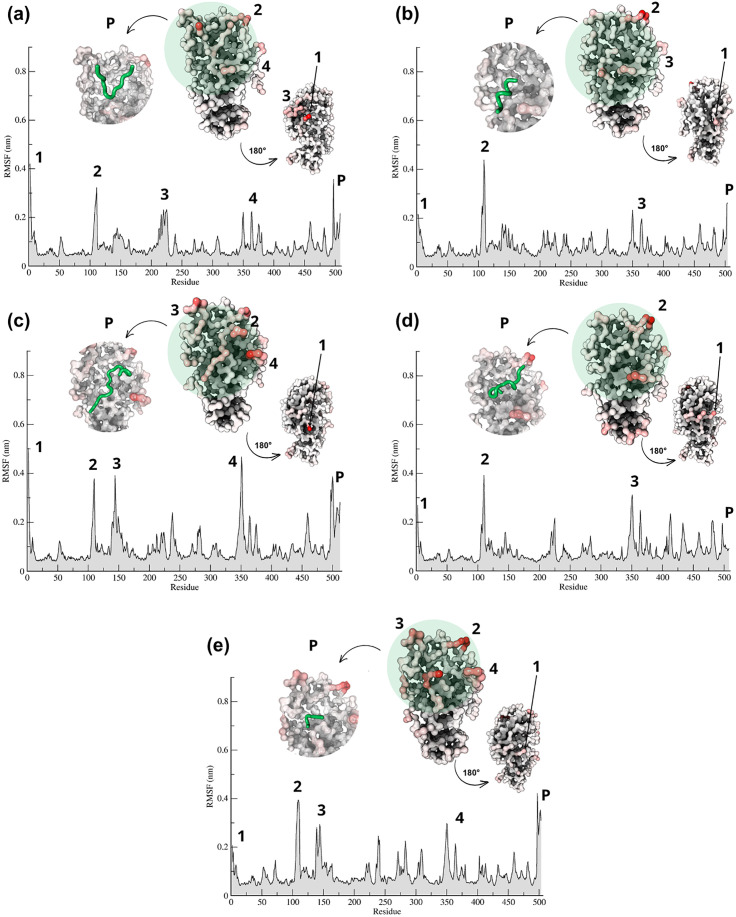
RMSF and B-factor profiles of peptides-α-amylase complexes (a) DTVHDTDGQVPL, (b) ILPAQQGK, (c) TVSQTPIDIPIGLPVR, (d)TIAPACAPKPAR and (e) DEQSSEK. Each panel shows the RMSF plot, where the peaks represent the fluctuations of each residue throughout the simulation, highlighting the peptide (P), and a surface representation of the B-factor highlighting the rotated structure of the enzyme (on the right) and the peptide in shades of green at the interaction site (P, on the left). The peptides are highlighted in green, and α-amylase in gray with red. Letters: D—Aspartic acid, E—Glutamic acid, R—Arginine, K—Lysine, N—Asparagine, H—Histidine, Q—Glutamine, S—Serine, T—Threonine, A—Alanine, G—Glycine, V—Valine, P—Proline, L—Leucine, I—Isoleucine, W—Tryptophan, C—Cysteine. The peptides are derived from the cleavage of TTIp 56/287: model number 56 and conformation number 287 of trypsin inhibitor purified from tamarind seeds.

In addition to the RMSD, the RMSF data (Fig 7) allowed us to evaluate the local conformational fluctuation of the complexes. The RMSF plot showed that the peptides TIAPACAPKPAR (Fig 7d) and ILPAQQGK (Fig 7b) presented the highest rigidity, followed by DTVHDTDGQVPL (Fig 7a). From RMSF, it can be observed that the conformational fluctuations remained within a range of up to 0.4 nm.

**Fig 8:**
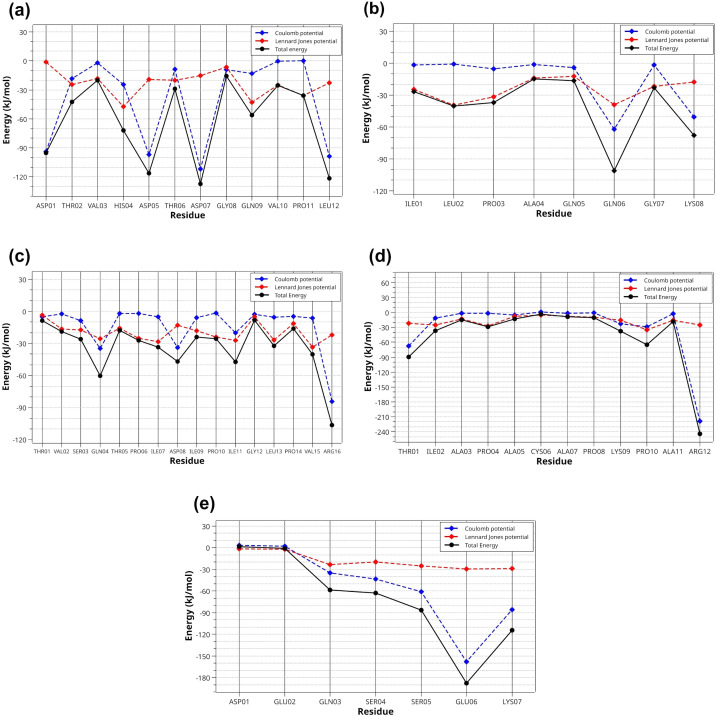
Interaction Potential Energy (IPE) Profiles per residue for the five peptides. (a) DTVHDTDGQVPL, (b) ILPAQQGK, (c) TVSQTPIDIPIGL, (d) TIAPACAPKPAR, and (e) DEQSSEK, complexed with the α-amylase enzyme, obtained from the last 50 ns of molecular dynamics simulation. The lines represent the Coulomb potential (blue), the Lennard-Jones potential (red), and the total energy (black) for each residue in the interaction site. Letters: D—Aspartic acid, E—Glutamic acid, R—Arginine, K—Lysine, N—Asparagine, H—Histidine, Q—Glutamine, S—Serine, T—Threonine, A—Alanine, G—Glycine, V—Valine, P—Proline, L—Leucine, I—Isoleucine, W—Tryptophan, C—Cysteine. The peptides are derived from the cleavage of TTIp 56/287: three-dimensional model number 56 and conformation number 287 of trypsin inhibitor purified from tamarind seeds.

A comprehensive analysis of intermolecular interactions between the peptides and α-amylase was conducted, yielding detailed Interaction Potential Energy (IPE) data (Fig 8). Interaction potential energies (IPE) were calculated from the sum of short-range Coulomb and Lennard-Jones potentials using data collected over the final 50 ns of the simulation (Table 3). The IPE values demonstrated that longer-chain peptides exhibited stronger attractive energies. Specifically, the peptide TVSQTPIDIPIGLPVR showed the most negative total energy, at −824.53 ± 74.43 kJ/mol. This was followed by DTVHDTDGQVPL, with a total energy of −705.08 ± 43.24 kJ/mol, and TIAPACAPKPAR, which presented −584.11 ± 53.26 kJ/mol. The Coulomb energy component exhibited greater variability across the complexes compared to the Lennard-Jones component. This suggests that long-range electrostatic interactions play a crucial role in stabilizing the peptides at the enzymatic binding site. Notably, complexes TVSQTPIDIPIGLPVR and DTVHDTDGQVPL displayed significant Coulomb potentials of −528.21 ± 74.43 kJ/mol and −410.41 ± 43.24 kJ/mol, respectively.

**Table 3:** Potential Interaction Energy for the peptide-α-amylase complexes for the last 50 ns of molecular dynamics simulation.

Peptide (Amino acid sequence)	Coulomb (kJ/mol)	Lennard-Jones (kJ/mol)	Total (kJ/mol)
DTVHDTDGQVPL	−410.41 ± 43.24	−294.67 ± 19.51	−705,08 ± 43,24
TIAPACAPKPAR	−379.70 ± 53.26	−204.41 ± 19.06	−584.11 ± 53.26
TVSQTPIDIPIGLPVR	528.21 ± 74.43	296.32 ± 25.69	−824.53 ± 74.43
DEQSSEK	−366.93 ± 40.61	−129.50 ± 16.68	−496.43 ± 40.61
ILPAQQGK	−149.51 ± 41.88	−204.78 ± 15.22	−354.29 ± 41.88

Letters: D—Aspartic acid, E—Glutamic acid, R—Arginine, K—Lysine, N—Asparagine, H—Histidine, Q—Glutamine, S—Serine, T—Threonine, A—Alanine, G—Glycine, V—Valine, P—Proline, L—Leucine, I—Isoleucine, W—Tryptophan, C—Cysteine. The peptides are derived from the cleavage of TTIp 56/287: three-dimensional model number 56 and conformation number 287 of trypsin inhibitor purified from tamarind seeds.

Analysis of the interaction potential energy profiles (Fig 8) indicated a clear predominance of Coulombic interactions (blue line), reflecting the electrostatic potential, over Lennard-Jones interactions (red line), which characterized van der Waals forces. At the binding site, these forces contributed differentially to the stability of the complexes, varying in intensity according to the participating residues. The black line indicates the total interaction energy, which is the sum of the Coulomb and Lennard-Jones interactions.

**Fig 9:**
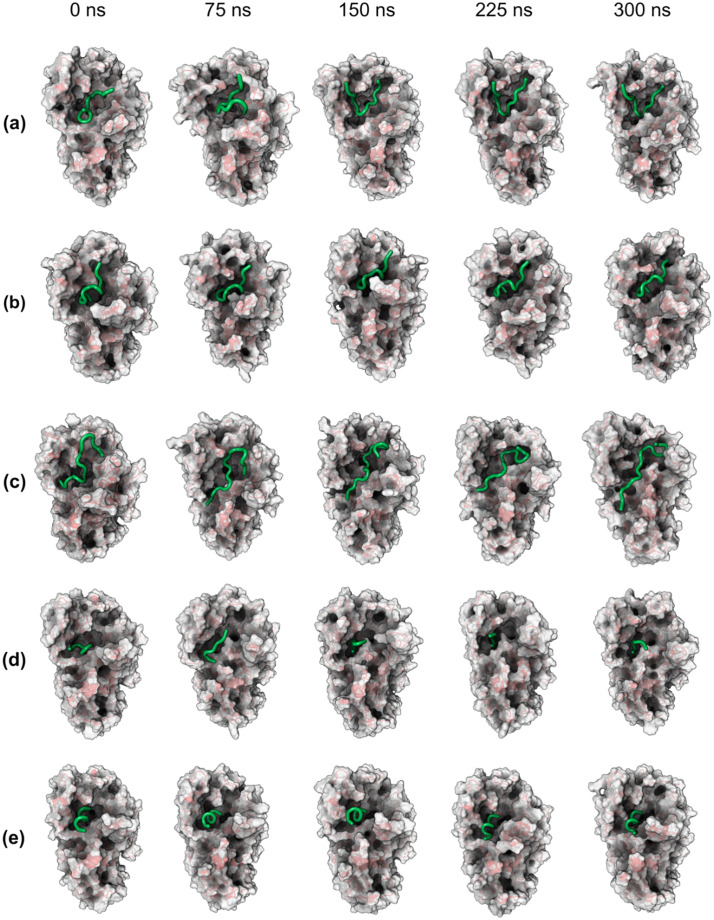
Three-dimensional cartoon and surface representations of the evolution of 300 ns of simulation. Configurations generated by molecular dynamics of the most stable complexes from molecular docking between α-amylase (PDB ID 5VA9) and the peptides (a) DTVHDTDGQVLP, (b) ILPAQQGK, (c) TVSQTPIDIPIGLPVR, (d) TIAPACAPKPAR and (e) DEQSSEK. Letters: D—Aspartic acid, E—Glutamic acid, R—Arginine, K—Lysine, N—Asparagine, H—Histidine, Q—Glutamine, S—Serine, T—Threonine, A—Alanine, G—Glycine, V—Valine, P—Proline, L—Leucine, I—Isoleucine, W—Tryptophan, C—Cysteine. The peptides are derived from the cleavage of TTIp 56/287: model number 56 and conformation number 287 of trypsin inhibitor purified from tamarind seeds.

**Fig 10:**
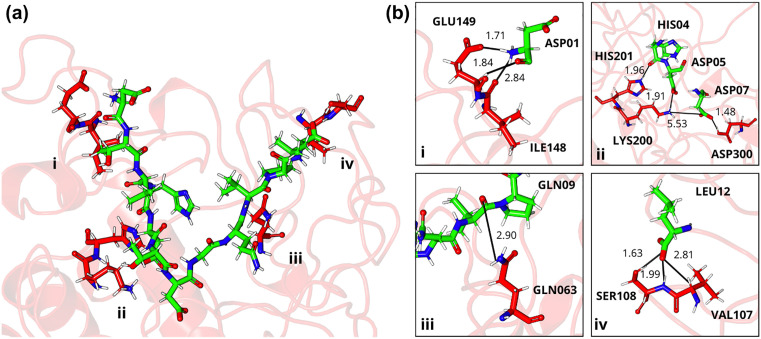
Three-dimensional stick model representation of the DTVHDTDGQVPL-α-amylase complex. Peptide illustrated in green and enzyme residues in red: (a) the interaction site highlighting key regions that promote complex stabilization and (b) electrostatic interactions between peptide-enzyme residue pairs, including charge-charge interactions (salt bridges) between ASP01-GLU149, ASP05-LYS200 and ASP07-LYS200; conventional hydrogen bonds between HIS04-HIS201, GLN09-GLN063 and LEU12-SER108; and hydrogen-carbon bond between ASP01-GLU149. Letters: D—Aspartic acid, E—Glutamic acid, R—Arginine, K—Lysine, N—Asparagine, H—Histidine, Q—Glutamine, S—Serine, T—Threonine, A—Alanine, G—Glycine, V—Valine, P—Proline, L—Leucine, I—Isoleucine, W—Tryptophan, C—Cysteine. The peptides are derived from the cleavage of TTIp 56/287: model number 56 and conformation number 287 of trypsin inhibitor purified from tamarind seeds.

**Fig 11:**
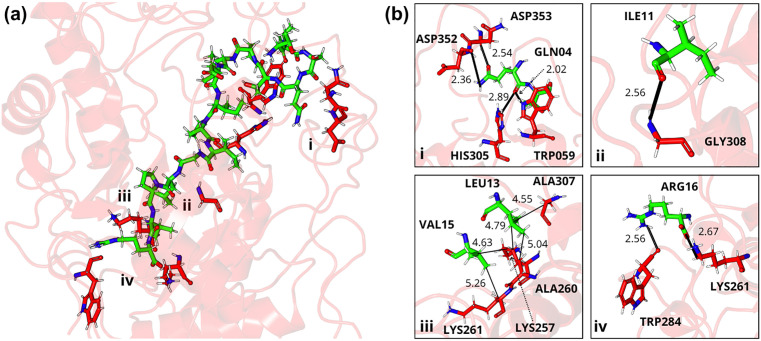
Three-dimensional stick model representation of the TVSQTPIDIPIGLPVR-α-amylase complex. Peptide illustrated in green and enzyme residues in red: (a) the interaction site highlighting key regions that promote complex stabilization and (b) electrostatic and van der Waals interactions between peptide-enzyme residue pairs, including conventional hydrogen bonding interactions between GLN04-TRP059, GLN04-ASP353, ILE11-GLY308, and ARG16-TRP284; hydrogen-carbon bonding interactions between GLN04-HIS305, GLN04-ASP352, and ARG16-LYS261; and alkyl-type interactions between LEU13-LYS257, LEU13-ALA260, VAL15-LYS257, and VAL15-LYS261. Letters: D—Aspartic acid, E—Glutamic acid, R—Arginine, K—Lysine, N—Asparagine, H—Histidine, Q—Glutamine, S—Serine, T—Threonine, A—Alanine, G—Glycine, V—Valine, P—Proline, L—Leucine, I—Isoleucine, W—Tryptophan, C—Cysteine. The peptides are derived from the cleavage of TTIp 56/287: model number 56 and conformation number 287 of trypsin inhibitor purified from tamarind seeds.

**Fig 12:**
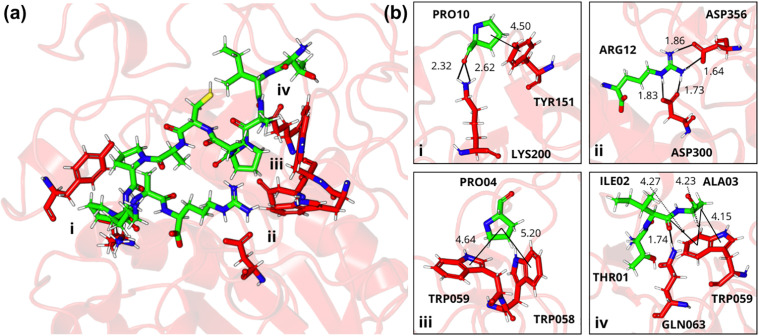
Three-dimensional stick model representation of the TIAPACAPKPAR-α-amylase complex. Peptide illustrated in green and enzyme residues in red: (a) the interaction site highlighting key regions that promote complex stabilization and (b) electrostatic and van der Waals interactions between peptide-enzyme residue pairs, including conventional hydrogen bonding interactions between PRO10-LYS200, ILE02-GLN063, and ARG12-ASP300; amide-pi stacking between ILE02-TRP059; pi-alkyl bonds between PRO10-TYR15, ALA03-TRP059, PRO04-TRP058, and PRO04-059; and salt bridge interactions between ARG12-ASP300 and ARG12-ASP356. Letters: D—Aspartic acid, E—Glutamic acid, R—Arginine, K—Lysine, N—Asparagine, H—Histidine, Q—Glutamine, S—Serine, T—Threonine, A—Alanine, G—Glycine, V—Valine, P—Proline, L—Leucine, I—Isoleucine, W—Tryptophan, C—Cysteine. The peptides are derived from the cleavage of TTIp 56/287: model number 56 and conformation number 287 of trypsin inhibitor purified from tamarind seeds.

The temporal evolution of the three-dimensional structures of the five α-amylase-peptide complexes across a 300 ns molecular dynamics simulation, with snapshots provided at 75 ns intervals, was demonstrated (Fig 9). The final conformational snapshots of peptides DTVHDDTDGQVPL, TVSQTPIDIPIGLPVR, and TIAPACAPKPAR, as determined by molecular dynamics simulations (Figs 10–12, respectively, using stick model representations). These figures showcase the peptides (green) positioned within the α-amylase interaction pocket alongside the interacting enzyme residues (red). The key interaction regions were further detailed [Figs 10b–12b, segmented into four distinct areas (i-iv)].

Analysis of the DTVHDDTDGQVPL-α-amylase complex (Fig 10a) revealed significant Coulomb interactions, including charge-charge (salt bridges) and hydrogen bonds. Residues ASP01 (−95.17 kJ/mol), HIS04 (−71.87 kJ/mol), ASP05 (−116.26 kJ/mol), ASP07 (−127.19 kJ/mol), and LEU12 (−121.60 kJ/mol) exhibited the highest energetic contributions to the intermolecular stability of the complex. Fig 10b presents specific intermolecular interactions. In region i, ASP01 (peptide) formed a 1.71 Å salt bridge with ILE148 (enzyme) and a 2.84 Å hydrogen bond with GLU149 (enzyme), while. ASP01-ILE148 pair formed a conventional 1.84 Å hydrogen bond. In region ii, the peptide interacted with the enzyme through residues HIS04, ASP05, and ASP07, maintaining a conventional hydrogen bond between the peptide-enzyme residue pairs HIS04-HIS201 (1.96 Å) and ASP07-ASP300 (1.48 Å); as well as charge-charge interactions between ASP05-LYS200 and ASP07-LYS200 (1.91 and 5.53 Å). In regions iii and iv, conventional hydrogen bonds were detected between the residue pairs GLN09-GLN063 (2.90 Å) and LEU12-SER108 (1.63 and 1.99 Å), respectively, and a hydrogen-carbon bond between LEU12-VAL107 (2.81 Å).

In the TVSQTPIDIPIGLPVR-α-amylase complex (Fig 11a), key stabilizing residues were identified, with ARG16 exhibiting the highest interaction energy (−106.63 kJ/mol), followed by GLN04 (−60.39 kJ/mol), ASP08 (−46.76 kJ/mol), ILE11 (−47.29 kJ/mol), VAL15 (−40.06 kJ/mol) and LEU13 (−32.31 kJ/mol).

Region i, illustrates the primary attractive interactions that stabilized the peptide-enzyme complex (Fig 11b). Specifically, conventional hydrogen bonds are observed between GLN04 and TRP059 (2.02 Å) and GLN04 and ASP353 (2.54 Å), while carbon-hydrogen bonds were formed between GLN04 and HIS305 (2.89 Å) and GLN04 and ASP352 (2.36 Å). Panels ii and iv highlight crucial interactions in this area of the pocket, such as conventional hydrogen bonds maintained between the pairs ILE11-GLY308 (2.56 Å) and ARG16-TRP284 (2.56 Å), in addition to the hydrogen-carbon bond ARG16-LYS261 (2.67 Å). Region iii was characterized by van der Waals interactions and alkyl-type interactions between the residue pairs LEU13-LYS257 (4.79 Å), LEU13-ALA260 (4.55 Å), VAL15-LYS257 (4.63 Å), and VAL15-LYS261 (5.26 Å).

In the TIAPACAPKPAR-α-amylase complex, key interactions included: a pi-alkyl interaction between PRO10 and TYR151; a conventional hydrogen bond between PRO10 and LYS200; a salt bridge (1.73 Å) and a conventional hydrogen bond (1.83 Å) between ARG12 and ASP300. Besides, two salt bridges between ARG12 and ASP356; pi-alkyl interactions between PRO04 and TRP058, and between PRO04 and TRP059; a conventional hydrogen bond between ILE02 and GLN063; amide-pi stacking between ILE02 and TRP059; and two pi-alkyl interactions between ALA03 and TRP059 (Fig 12a).

It is crucial to consider the relevant influence of electrostatic interactions in the case of salt bridges in the TIAPACAPKPAR-α-amylase complex. The electrostatic interactions mediated by the ARG12 residue of the peptide and the ASP300 and ASP356 residues of the enzyme played a crucial role in stabilizing the salt bridges within the complex, leading to a substantial reduction in the complex’s potential energy, measured at −244.23 kJ/mol (Fig 12b).

The computational prediction of the bioactivity of the selected peptides was also analyzed (Table 4). The peptides TIAPACAPKPAR and DTVHDTDGQVPL stood out in terms of bioactive potential (PeptideRanker) and half-life analyses, demonstrating high stability.

**Table 4:** Computational prediction of bioactive potential, cell penetration capacity, half-life in the simulated intestinal environment, and peptide stability.

Peptide (Amino acid sequence)	PeptideRanker Score	CPP potential	Half-life (seconds)	Stability
DTVHDTDGQVPL	0.1047730	No-PPC	1.444	High
TIAPACAPKPAR	0.6096820	No-PPC	1.835	High
TVSQTPIDIPIGLPVR	0.2720040	No-PPC	0.675	Normal
DEQSSEK	0.0487441	No-PPC	–	–
ILPAQQGK	0.2237500	No-PPC	–	–

Peptides were generated by theoretical cleavage of purified trypsin inhibitor from tamarind seeds (TTIp) [model number 56, conformation number 287] (TTIp 56/287) with the enzyme’s chymotrypsin and trypsin in combination. PeptideRanker: predicts the predicted probability of a peptide being bioactive. CellPPD: ranks the peptide by its probability of cell penetration. CPP: cell-penetrating peptides. HLP: predicts the half-life of peptides in a gut-like environment (the software does not support peptides <10 aa). Letters: D—Aspartic acid, E—Glutamic acid, R—Arginine, K—Lysine, N—Asparagine, H—Histidine, Q—Glutamine, S—Serine, T—Threonine, A—Alanine, G—Glycine, V—Valine, P—Proline, L—Leucine, I—Isoleucine, W—Tryptophan, C—Cysteine. The peptides are derived from the cleavage of TTIp 56/287: three-dimensional model number 56 and conformation number 287 of trypsin inhibitor purified from tamarind seeds.

## 4. Discussion

The search for therapeutic alternatives for the management of Diabetes Mellitus (DM) has generated intense interest due to the impact of this disease, especially when related to persistent hyperglycemia [37]. Several approaches have been studied, including blocking or inhibiting therapeutic targets such as α-amylase, an enzyme responsible for carbohydrate digestion. In this context, peptide drugs have gained prominence due to their greater potency, tissue specificity, and reduced side effects [38]. For example, between 2015 and 2019, the Food and Drug Administration (FDA) approved 15 peptide drugs, corresponding to 7% of the total approvals, thereby reinforcing the potential of these compounds in modern therapeutics [39].

Recent advances in peptide screening and computational biology approaches have facilitated the development of new peptide drugs, allowing the screening and identification of active molecules through bioinformatics tools [23,37]. Many studies performed in silico have been subsequently confirmed in vivo, reinforcing the validity of computational findings [23,37]. Thus, the results of the present study were presented with the perspective that the trypsin inhibitor isolated from tamarind seed (*Tamarindus indica* L.), named TTI, could be a potential candidate for inhibiting α-amylase. In this study, TTI and especially two TTI-derived peptides, in vitro and in silico, showed the highest potential to inhibit α-amylase.

The obtaining of TTI was reproduced and confirmed through 12% SDS-PAGE stained with Coomassie Brilliant Blue G250, revealing protein bands with a predominance of molecular mass around 21 kDa. These data on the isolation and characterization of TTI agree with previous studies, such as those carried out by Carvalho et al. (2016) [15], Costa et al. (2018) [40], Costa et al. (2022) [41], and Lima et al. (2022) [42]. After obtaining, the in vitro α-amylase inhibition test was performed, demonstrating an inhibition of 37.3%. Related studies using seed protein hydrolysates, such as those from Luffa cylindrica [43], revealed inhibitions of up to 28% (when cleaved with trypsin) and 25% (with pepsin). In comparison, Awosika and Aluko (2019) [44] reported an inhibitory activity of 30.39% with yellow pea (*Pisum sativum* L.) protein hydrolysates. Thus, a similarity is observed between the inhibitory percentages found in these studies and the value obtained for the TTI. Furthermore, in this in vitro study, TTI was isolated and not completely purified.

Although acarbose, a purified synthetic inhibitor specific for α-amylase, presents up to 100% inhibition, TTI, a multifunctional natural protein with 100% antitryptic activity, achieved almost 40% inhibition. This fact sparked interest in investigating TTI-derived peptides, as it is known that isolated or purified peptides tend to bind more easily to the enzyme’s active site due to their size and more intense chemical interactions. In contrast, hydrolysates contain a wide variety of peptides and present weaker enzymatic binding capacity [38].

If TTI were purified, it would likely have exhibited greater affinity for α-amylase and, consequently, a higher inhibition rate. However, purified TTI has a molecular weight of 19 kDa [14]. Therefore, prospecting for TTI-derived peptides with affinity for α-amylase appears to be a more promising strategy.

Given this perspective, the study investigated, through computational tools, the interactions between the theoretical TTI (TTIp 56/287) and α-amylase using molecular modeling techniques. Ten different docking sequences were suggested to elucidate conformations with higher binding affinities. These analyses revealed that variations of TTIp 56/287 in three-dimensional space resulted in the identification of specific domains that demonstrate enhanced interaction with the target enzyme. This variety of docking possibilities reinforced the strategy of prospecting peptides derived from TTIp 56/287 as potential α-amylase inhibitors.

The molecular docking methodologies employed, notably through the HADDOCK 2.4 server, identified the preferred conformations of the complexes formed between TTIp 56/287 and α-amylase. And between the derived peptides and the enzyme. In docking studies for macromolecules, such as proteins, it is essential to previously orient an active region for anchoring in the binding site [44].

Based on the scoring results, the conformation generated using sequence 2 (PLNNAGQYYI) demonstrated significantly greater stability, indicated by the highest HS value, followed by the conformation derived from sequence 1 (DTVHDTDGQV). These data suggest that the derived peptides have an affinity for α-amylase, forming stable and energetically favorable interactions. Based on these results, five peptides derived from TTIp 56/287, previously indicated by Gomes et al. [23], were selected so that their complexes with α-amylase could be subjected to molecular dynamics simulations and the binding free energy calculation.

Molecular dynamics simulations were performed for 300 ns for each of the five peptide-α-amylase complexes (using the structure of human pancreatic α-amylase, PDB ID: 5VA9). Analysis of the RMSD throughout the simulation revealed that, after an initial period of significant fluctuations, the values stabilized, ranging from 0.08 to 0.3nm, after approximately 100 ns. This stabilization indicates that the systems reached a state of dynamic equilibrium, which confers reliability to the conformations sampled in the last 100 ns for subsequent analyses, such as calculating the binding free energy [45,46].

The RMSF analysis highlighted that the peptides TIAPACAPKPAR and ILPAQQGK were the most stable, in terms of rigidity, followed by DTVHDTDGQVPL. These three peptides showed a greater propensity to form hydrogen bonds [45]. While TIAPACAPKPAR showed a slightly better structural alignment with reference conformation, DTVHDTDGQVPL presented the best HS value, as indicated in Table 2. These observations highlight the complex interaction between hydrogen bond formation, structural deviations, and stability. This underscored the importance of visualizing the three-dimensional intermolecular interactions between peptides and α-amylase. It is worth noting that, in a study by Alhawday et al. (2024) [47], RMSF values for α-amylase inhibitory peptides reached up to 3.5 nm, suggesting that TTI-derived peptides 56/287 exhibit superior rigidity and stability in enzyme inhibition, which may consequently impact the inhibitory potential and activity of these peptides.

In the IPE analysis, the results indicated that the interaction energy is more evenly distributed along the peptide chain in the complexes formed by DTVHDTDGQVPL-α-amylase and TVSQTPIDIPIGLPVR-α-amylase. In contrast, other complexes, even without complete uniformity in the distribution of favorable interactions, showed residues with significant energy contributions, as observed in the TIAPACAPKPAR-α-amylase complex [45]. It is essential to consider that Coulomb interactions, of the charge-charge type and the salt bridges formed between charged amino acids (such as ARG, ASP, GLU, HIS, and LYS), played a crucial role in the stabilization of ligand-protein complexes together with conventional hydrogen bonds and hydrogen-carbon bonds.

Furthermore, mapping of the enzyme residues most involved in the interactions highlighted the key roles of GLN036, SER108, GLU149, LYS200, ASP300, ASP352, ASP353, ASP356, ARG195, HIS305, and GLY304. These findings corroborate previous studies that employed in silico approaches to investigate peptides derived from natural or non-natural sources with inhibitory potential against α-amylase [44,47]. Based on the study of the interaction energies per residue, it is proposed to modify the polarity of the central regions of DTVHDTDGQVPL and TIAPACAPKPAR to maximize the attractive intermolecular interactions in these regions, thereby conferring a greater binding affinity toward α-amylase.

Unlike previous studies that focused solely on molecular docking, the present research advanced the analysis by employing molecular dynamics simulations, extending up to 300 ns for each peptide-α-amylase complex. This approach enabled a detailed investigation of the key amino acid residues in the enzyme’s catalytic site, which are critical for substrate binding. The insights gained from this analysis may contribute to the more efficient design of analogous peptides with enhanced α-amylase inhibitory activity [45].

Complementary studies, such as that of Alhawday et al. (2024) [47], who developed and evaluated isoxazolidine derivatives as potential α-amylase inhibitors, reinforced the importance of these approaches. In their work, the synthesized compounds demonstrated promising in vitro inhibitory activities, with IPE values below −400 kJ/mol, high inhibitory potency (approximately 50%), and stability, evidencing a correlation between in silico data and experimental results.

In the therapeutic context, the greater the inhibitory potential of a molecule on α-amylase, the greater its efficacy in treating hyperglycemia associated with DM [48]. Acarbose, although a synthetic inhibitor with excellent inhibitory results, is related to adverse effects such as abdominal pain, flatulence, and diarrhea. This limitation reinforces the need to seek therapeutic alternatives with a lower incidence of side effects [49].

In silico analyses of the bioactive potential revealed that DTVHDTDGQVPL and TIAPACAPKPAR peptides exhibited superior stability and a prolonged half-life compared to TVSQTPIDIPIGLPVR. In particular, the TIAPACAPKPAR peptide achieved the highest score on the PeptideRanker server (exceeding 0.6), standing out as the candidate with the greatest predicted bioactive potential. One of the challenges in applying peptides with biological action is the low stability during the digestive process, resulting from the action of intestinal proteases [50].

Therefore, a half-life prediction study was conducted in a simulated intestinal environment, where all the peptides analyzed showed normal or high stability. The stability of peptides in a simulated intestinal environment is crucial for oral applications. It is rarely evaluated in initial peptide prospecting studies, thereby serving as a differentiator of this study. However, assessing peptides with fewer than 10 amino acids was impossible. This limitation is inherent to the tool used, and therefore, more robust analyses are necessary in the future to confirm the data presented. To date, there are no studies in the literature that confirm the reliability of these predictors for α-amylase inhibitory peptides or whether similar predictions have been experimentally validated in prior literature.

In summary, the prospecting of bioactive TTI-derived peptides has proven to be a promising alternative for treating T2DM, as it effectively inhibits α-amylase from a natural trypsin inhibitor source. The data indicated that the TTIp 56/287 complex (evaluated in vitro and silico) and the peptides DTVHDTDGQVPL and TIAPACAPKPAR (evaluated in silico) interact effectively with the enzyme. These peptides were assessed in silico, without an assessment of toxicity, immunogenicity, or off-target effects. In addition, none were tested in vitro or in vivo. Therefore, the peptide synthesis, possible delivery systems, optimization of peptide length, and its action on α-amylase must be biologically validated in the future.

These results, following in vitro and in vivo validation, will confirm whether these peptides might reduce dosing frequency, whether there is a risk of non-specific inhibition of other digestive enzymes, and potential effects on postprandial glucose curves compared to existing drugs. Therefore, this study, still hypothetically, suggests the potential of these hydrolysates to be used as functional ingredients in the development of nutraceutical products or even drugs intended for the control and treatment of T2DM, offering an alternative with a lower risk of adverse effects compared to the synthetic inhibitors currently available.

## 5. Conclusion

The present study evaluated the in vitro inhibitory and interaction effects of TTI and/or its peptides on α-amylase. Regarding computational analyses, the docking and molecular dynamics simulation results showed that TTIp 56/287 and its peptides interacted stably and with affinity with the α-amylase molecule.

From the RMSD, RMSF, and IPE, it was observed that the peptides DTVHDTDGQVPL and TIAPACAPKPAR demonstrated the most promising results. This observation was further supported by bioactivity, which revealed the high structural stability of these two molecules. Therefore, these peptide sequences are highlighted as promising candidates for future studies aimed at validating their hypoglycemic potential through α-amylase inhibition, along with an acceptable safety profile. Consequently, they are excellent candidates for future in vitro and in vivo analyses.

## Supporting information

S1 File Supporting information. (ZIP)
